# Impact of Pulmonary Arteriovenous Malformations on Respiratory–Related Quality of Life in Patients with Hereditary Haemorrhagic Telangiectasia

**DOI:** 10.1371/journal.pone.0090937

**Published:** 2014-03-06

**Authors:** Sandra Blivet, Daniel Cobarzan, Alain Beauchet, Mostafa El Hajjam, Pascal Lacombe, Thierry Chinet

**Affiliations:** 1 Aphp, Université De Versailles Saint Quentin En Yvelines, Consultation Pluridisciplinaire Maladie De Rendu-Osler, Hôpital Ambroise Paré, Boulogne, France; 2 Aphp, Université De Versailles Saint Quentin En Yvelines, Département De Santé Publique, Hôpital Ambroise Paré, Boulogne, France; Odense University Hospital, Denmark

## Abstract

Fifteen to fifty percent of patients with hereditary haemorrhagic telangiectasia have pulmonary arteriovenous malformations. The objective of this study was to measure the effect of the presence of pulmonary arteriovenous malformations and of their embolisation on respiratory-related quality of life (QoL). We prospectively recruited patients with a diagnosis of hereditary haemorrhagic telangiectasia based on the Curaçao criteria and/or the identification of a pathogenic mutation. Respiratory-related quality of life was measured using the Saint George’s Respiratory Questionnaire (SGRQ). Patients who underwent embolisation of pulmonary arteriovenous malformations completed the questionnaire before and 6–12 mo after the procedure. The 56 participants were divided into three groups: no pulmonary arteriovenous malformation (group A, *n* = 10), small pulmonary arteriovenous malformations not accessible to embolotherapy (group B, *n* = 19), and large pulmonary arteriovenous malformations accessible to embolotherapy (group C, *n* = 27). The SGRQ score was significantly higher in group C compared to the other groups, indicating a worse respiratory-specific QoL. There was no significant difference between groups A and B. Among the 17 patients who underwent an embolisation, the SGRQ score decreased significantly after the procedure, to a value similar to that in patients without pulmonary arteriovenous malformation. Our results indicate that the presence of large but not small pulmonary arteriovenous malformations negatively affects the respiratory-related quality of life and that embolisation of pulmonary arteriovenous malformations normalizes the respiratory-related quality of life.

## Introduction

Hereditary haemorrhagic telangiectasia (HHT), also known as Rendu-Osler-Weber syndrome, is a genetic vascular disorder with an autosomal dominant inheritance pattern. Its prevalence is estimated at around 1∶5,000–1∶10,000 [1], [2]. The clinical manifestations of HHT include epistaxis; telangiectasia on the skin and mucosal membrane; and visceral arteriovenous malformations (AVMs), predominantly in the lung, brain, gastrointestinal tract, and liver. HHT is associated with mutations in genes involved in the transforming growth factor β (TGF-β) signaling pathway [3]. Mutations in three genes have been found to be responsible for HHT: the endoglin (*ENG*) gene on chromosome 9, the activin-receptor-like kinase 1 (*ACVRL1*) gene on chromosome 12, and the *SMAD4* gene on chromosome 18 (mutations also lead to juvenile polyposis). Other loci have been identified, but the genes have not yet been discovered. The phenotypic expression of the disease depends on the mutated gene: for instance, patients with *ENG* mutations (“HHT1 phenotype”) are more likely to have pulmonary arteriovenous malformations (PAVMs) than patients with *ACVRL1* mutations (“HHT2 phenotype”). In addition, clinical expression of the disease usually worsens with age. A diagnosis of HHT is established based on the Curaçao criteria (nose bleeds, mucocutaneous telangiectasia, visceral arteriovenous malformations, and family history) or on the identification of a pathogenic mutation [2], [4].

PAVMs are found in approximately 15–50% of HHT patients [1]–[4]. The presence of PAVMs exposes the patient to significant clinical risks. PAVMs constitute a right-to-left shunt, leading to hypoxaemia that is poorly responsive to supplemental oxygen. Rupture of the thin wall of the PAVM sac in the airways or pleural cavity may cause haemoptysis or haemothorax [5]. In addition, the shunting of the pulmonary capillary bed may allow thrombi, bacteria, and air bubbles to reach the systemic circulation, mainly the cerebral circulation, resulting in paradoxical systemic embolic complications. Cerebral vascular complications (i.e., stroke and transient ischemic attack) and abscesses occur in approximately 30% of patients with PAVMs [5]–[7]. Management of PAVMs includes prophylactic administration of antibiotics before dental and surgical procedures and embolisation. The treatment of choice for PAVMs is percutaneous transcatheter embolisation of the feeding arteries, which improves blood oxygenation and decreases the risk of paradoxical embolic stroke and cerebral abscess [4], [5], [6], [8]. Embolisation is usually a safe procedure when performed by expert operators. It is recommended to patients with HHT when PAVMs accessible to the procedure are found, even when patients are asymptomatic. Long-term clinical success for embolisation in patients with PAVMs has been extensively reported [4]–[6], [8], [9].

Several studies have shown that HHT negatively impacts quality of life (QoL) [10]–[14]. One of the most significant clinical manifestations associated with poor QoL is severe epistaxis. However, these studies focused on overall, health-related, and symptom-specific aspects of QoL. Since PAVMs have a substantial clinical impact on patients with HHT, we focused on the impact of PAVMs on QoL in patients with HHT and evaluated the effect of embolisation of PAVMs on QoL.

## Materials and Methods

### Participants

All consecutive patients with HHT who presented to our center between January 2010 and January 2012 for evaluation of their disease were invited to participate in this prospective study. We also recruited patients who were admitted during the same period of time for embolisation of PAVM following evaluation of their disease during the previous year. To be included in the study, patients had to fulfill the following criteria: (1) diagnosis of HHT ascertained by identification of an *ENG*, *ACVRL1*, or *SMAD4* mutation and/or by the presence of at least three Curaçao criteria; (2) age >15 y; and (3) fluency in the French language (4). Patients were not included if they were suffering from chronic respiratory conditions unrelated to HHT. All participants signed an informed consent form. This study was declared to the Commission Nationale Informatique et Liberté (CNIL), was performed in accordance with French regulations and the Helsinki Declaration and was approved by the Société de Pneumologie de Langue Française (SPLF) institutional review board (CEPRO).

### Study Protocol

Our routine work-up for the evaluation of the disease included clinical examination by skin, ENT, neurology, pulmonary, and gastroenterology specialists; blood tests; genetic clinical evaluation and testing; chest and abdominal high-resolution CT scans; transthoracic cardiac echocardiography; cerebral magnetic imaging; and pulmonary function tests.

Patients who underwent embolisation of PAVMs were informed about the risks and benefits of the procedure and gave informed consent. The procedure was performed as previously reported [9].

### Quality of Life

Respiratory-specific QoL was evaluated with Saint George’s Respiratory Questionnaire (SGRQ) (www.healthstatus.sgul.ac.uk/sgrq). This questionnaire was self-administered to participants. The SGRQ measures three components: the “symptoms component” is concerned with the most common respiratory symptoms; the “activity component” is concerned with activities that cause or are limited by breathlessness; and the “impact component” is concerned with the perceived impact of respiratory symptoms on the patient’s social life and psychological status (15). The questionnaire has 50 items. The scale is scored from 0 to 100, where the higher score indicates worse health status and a difference or change of four points is considered clinically significant [16].

Patients were instructed to complete the questionnaire during their evaluation in the department or at the time of admission for the embolisation procedure. Patients who underwent embolisation were instructed to answer a second similar questionnaire approximately 6 mo after the procedure. The questionnaire was sent by mail approximately 5 mo after the procedure. If the patient failed to answer by 6 mo after the procedure, a second questionnaire was sent by mail 1–2 mo later. If the patient failed to answer the second mailing, contact was attempted by telephone.

### Data Analysis

Participants were divided into three groups: patients with no PAVM at chest CT scan (group A), patients with “small” PAVMs not accessible to embolotherapy because the diameter of the feeding arteries was too small (i.e., <2.5–3 mm; group B), and patients with “large” PAVMs accessible to embolotherapy (group C).

Quantitative data are expressed as mean ± standard deviation (SD) and range; qualitative data are expressed as frequency and percent. Comparisons of quantitative values were performed using the Wilcoxon rank sum test, the Wilcoxon signed-rank test and the Kruskal–Wallis test as appropriate. The Kruskal-Wallis test was completed in case of significance by multiple comparisons tests. Comparisons of frequencies were performed with the Chi-square test and the Fisher’s exact test as appropriate. A *p* value of <0.05 was considered statistically significant. Statistical analysis was performed with SAS software version 9.3 (SAS Institute Inc, Cary, NC, USA).

## Results

### Characteristics of Patients

Fifty-seven patients with HHT were invited to participate in the study. One female patient declined the invitation. The 56 participants comprised 24 men and 32 women; their mean age was 42.5±17.2 y (range 16–80 y). Mutations in *ENG* were identified in 31 patients (55%), mutations in *ACVRL1* were identified in 17 patients (30%), and one patient had a mutation in *SMAD4* and presented with juvenile polyposis. No mutation was identified in seven patients (13%); these patients fulfilled three or four Curaçao criteria. All but one of the patients reported recurrent nosebleeds. Eight patients reported gastrointestinal bleeding. Hepatic expression of the disease was identified in 29 patients (52%). Four patients had cerebral vascular malformations, which had been treated by embolisation prior to this study in all cases. Five patients with PAVMs reported a history of cerebral complication of PAVMs (one abscess and four transient ischemic attacks). The average haemoglobin level was 13.8±2.1 g/dl (range 7.5–16.7 g/dl). The characteristics of the 56 participants according to their study group are reported in Table 1.

**Table 1:** Characteristics of the study participants.

	GROUP A	GROUP B	GROUP C	p-VALUE
**N**	10	19	27	
**Age (years)**	48.9±13.7	40.0±16.9	41.9±18.5	0.34
**Female sex, n (%)**	4 (40)	12 (63)	16 (59)	0.46
**Mutational status**				0.008
** ENG mutation**	1	11	19	
** ACVRL1 mutation**	8	6	3	
** MADH4 mutation**	0	1	0	
** No mutation**	1	1	5	
**Hb levels (g/dL)**	14.0±2.3	13.9±1.4	13.6±2.5	0.93
**O_2_ saturation (%)**	98±1	98±1	95±4	0.002
**Global score**	10.5±7.5	11.5±12.9	22.0±17.2	0.03
**«Symptoms» score**	9.4±10.5	7.2±10.2	9.6±8.1	0.50
**«Activity» score**	25.8±19.4	24.1±22.0	40.2±28.3	0.07
**«Impact» score**	2.1±2.7	5.6±11.7	15.4±18.2	0.01

O_2_ saturation was measured in sitting position while breathing room air.

Hb: haemoglobin.

### Evaluation of SGRQ

**Figure 1:**
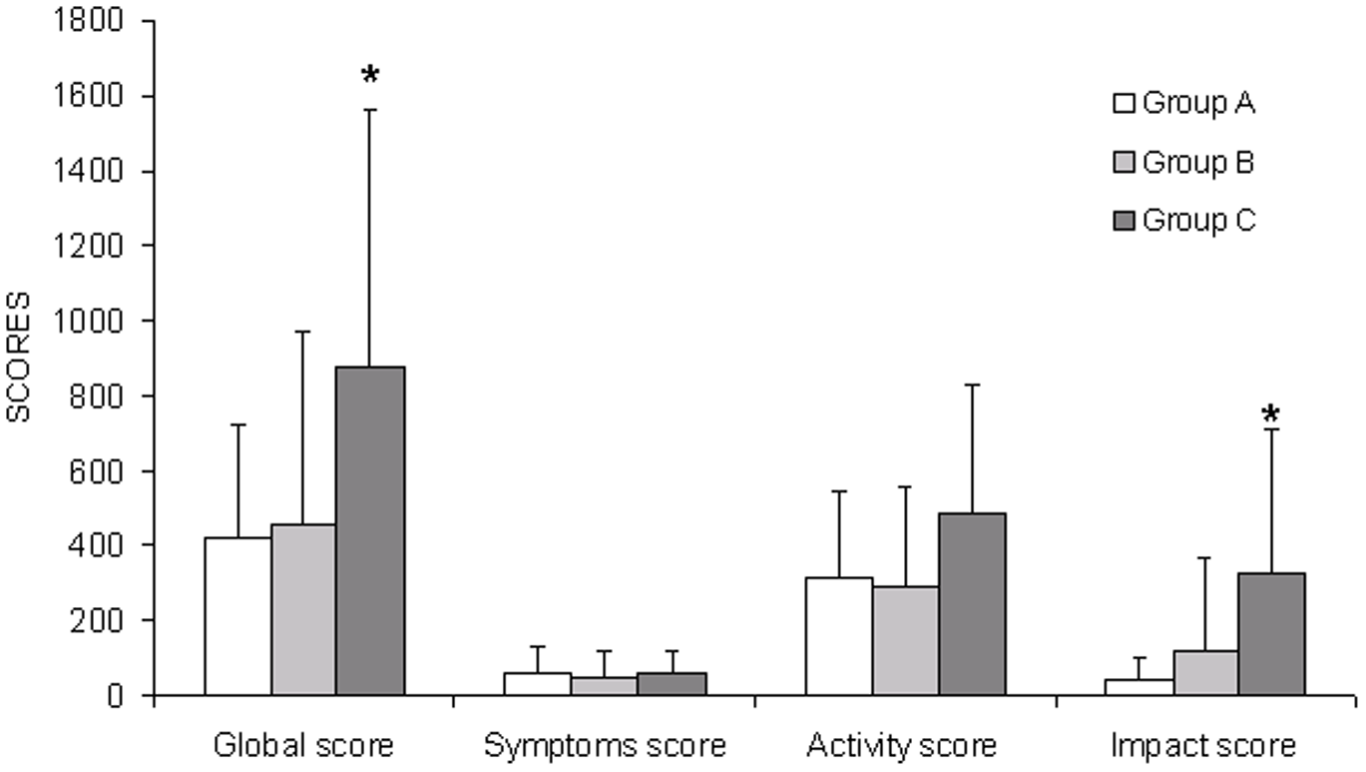
Global score of the Saint George’s Respiratory Questionnaire and score of its “symptoms” component, “activity” component and “impact” component in the 3 groups of patients: group A (patients with no PAVM), group B (patients with “small” PAVMs) and group C (patients with “large” PAVMs). Bars indicate standard deviation.

The global score for the SGRQ was significantly different between the three groups (Table 1 and Figure 1). The global score was significantly higher in group C compared to the other groups (*p* = 0.04 compared to group A and *p* = 0.02 compared to group B), indicating worse respiratory-specific QoL. There was no significant difference between groups A and B.

The score for the impact component was significantly higher in group C than in the other groups (*p* = 0.02 compared to group A and *p* = 0.007 compared to group B); there was no significant difference between groups A and B. We also found no difference among the groups in the score for the symptoms or activity components.

### Impact of Embolisation of PAVM on QoL

Eighteen patients underwent embolisation of PAVMs in our center at least 6 months prior to the end of the timeframe of the study. One patient declined to answer the second questionnaire. We therefore report the data for 17 patients. As shown in Table 2, there were no significant differences in the characteristics of these patients and the remaining ten patients of group C. Adverse events related to the procedure were documented in two patients: one transient ischemic attack and one thrombosis of a calf vein.

**Table 2:** Characteristics of the group C patients who participated in the evaluation of the impact of embolisation on the quality of life.

	PARTICIPANTS	NON-PARTICIPANTS	p-VALUE
N	17	10	
**Age (years)**	42.5±19.4	40.9±17.8	0.82
**Female sex, n (%)**	10 (59%)	6 (60%)	0.95
**Hb levels (g/dL)**	13.8±2.0	13.3±3.2	0.86
**O_2_ saturation (%)**	95±5	94±4	0.85
**Global score**	21.5±16.0	22.8±18.4	1.00
**«Symptoms» score**	7.6±7.5	13.1±8.3	0.24
**«Activity» score**	43.4±28.6	34.8±28.4	0.45
**«Impact» score**	13.4±17.7	19.0±19.3	0.35

O_2_ saturation was measured in sitting position while breathing room air.

Hb: haemoglobin.

**Figure 2:**
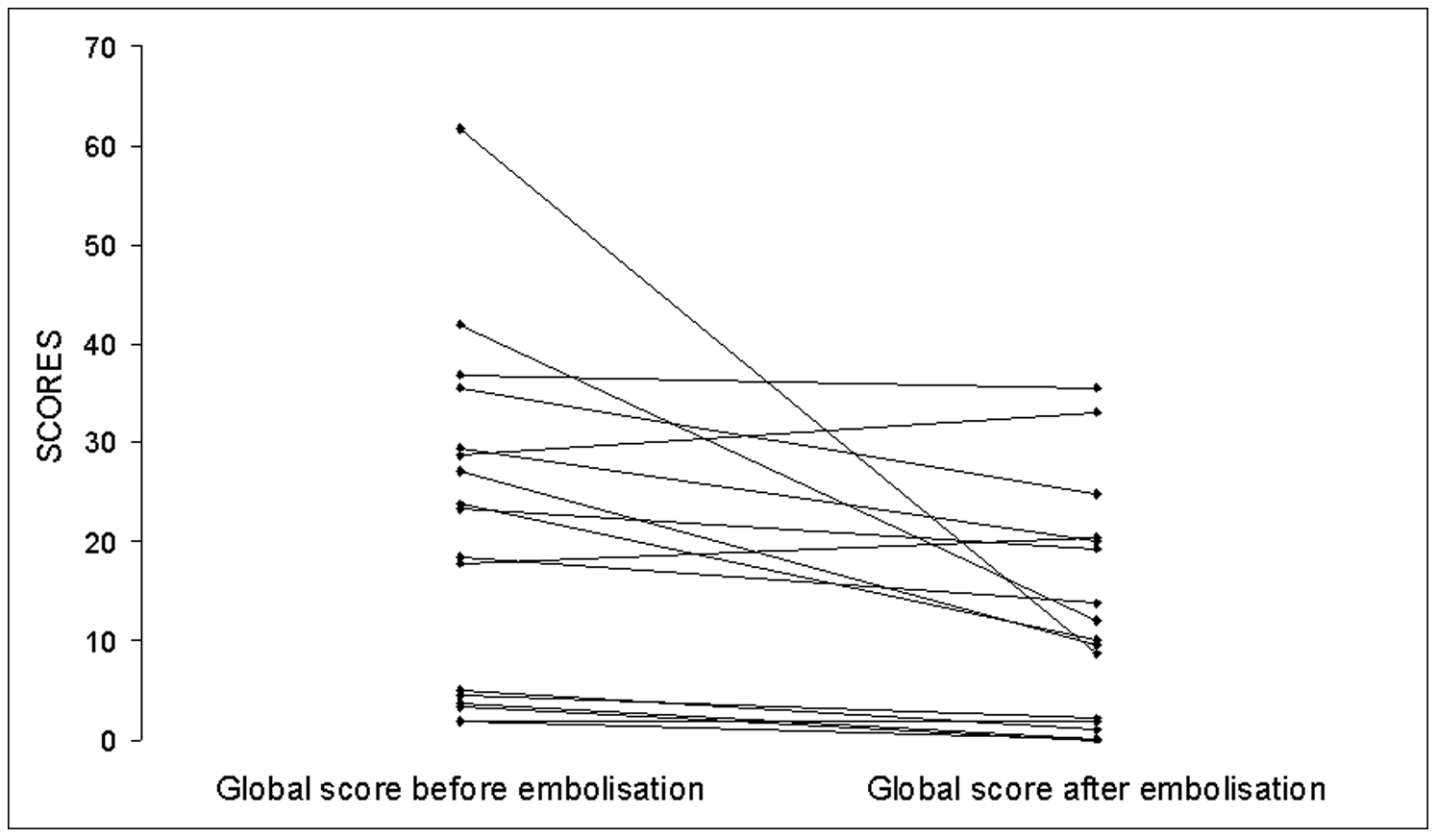
Global score of the Saint George’s Respiratory Questionnaire in 17 HHT patients before and 6–12 months after embolisation of PAVMs.

Both the global score and the score for the activity component decreased significantly after embolisation of PAVMs (*p* = 0.003 and *p* = 0.0006, respectively), whereas the score for the impact and symptoms components did not change significantly (Table 3 and Figure 2). We found no statistically significant differences for any of the four scores between group C after embolisation and groups A and B.

**Table 3:** Impact of embolisation on quality of life.

	BEFORE EMBOLISATION	AFTER EMBOLISATION	p-VALUE
**Global score**	21.5±17.0	12.5±11.5	0.003
**«Symptoms» score**	7.6±7.5	6.4±7.4	0.19
**«Activity» score**	43.4±28.6	25.9±23.5	0.0006
**«Impact» score**	13.4±17.7	6.8±8.6	0.11

## Discussion

Our results demonstrate that the presence of PAVMs significantly impairs the QoL of patients with HHT and that embolisation of PAVMs not only improves their respiratory-specific QoL but raises it to the same level as observed in HHT patients without PAVM (as measured with the SGRQ). In addition, the presence of “small” PAVM(s) (i.e., not accessible to embolisation) does not appear to affect the respiratory-specific QoL.

To our knowledge, this is the first study to examine the respiratory-specific health status of HHT patients. Health-related QoL has been measured before in HHT patients, but the impact of the presence of PAVMs on QoL has not been evaluated to date. Most studies on health-related QoL in HHT patients used a general health questionnaire such as the Short Form-36 (SF-36) questionnaire [10]–[14]. This questionnaire measures eight dimensions: role limitation due to physical health problems, physical functioning, role limitation due to emotional problems, bodily pain, general health, vitality, social functioning, and mental health. For instance, Geistholl UW et al. found that in 77 HHT patients, impairment of QoL, as measured by the SF-36 questionnaire, was associated with digestive bleeding and the severity of epistaxis [12]. The negative impact of severe epistaxis on QoL has been confirmed in other studies [10], [11], [13], [17], [18]. Interestingly, Pfister et al. reported that HHT2 patients have a higher health-related QoL than HHT1 patients and suggested that this might be due to the fact that 30–40% of HHT1 patients have PAVMs compared to 0–14% of HHT2 patients [14]. Our study confirms this hypothesis: in addition to other manifestations of HHT, such as severe epistaxis and digestive bleeding, the presence of PAVMs significantly impairs health-related QoL.

The presence of PAVMs predisposes patients to severe complications. It is therefore recommended that clinicians screen all HHT patients for the presence of PAVMs (e.g., by contrast echocardiography). In patients with PAVMs, an embolisation procedure should be performed whenever technically possible. Complications related to the procedure include device migration, stroke, gaseous embolism, pulmonary infarction and haemoptysis, and reperfusion of embolised PAVMs [4]–[6], [8]. However, the risks of procedure-related complications are considered to be outweighed by the benefits of embolotherapy when the procedure is performed in centers of excellence [4], [8]. Unfortunately, it is sometimes difficult to convince patients to undergo such a procedure when no complications due to PAVMs have yet occurred. Patients are usually more concerned with issues that have an immediate daily impact, such as epistaxis and gastrointestinal blood loss, than with theoretical risks from a PAVM, however potentially debilitating that risk might be. It is therefore crucial to help patients to understand the risks related to the presence of PAVMs [8]. In addition, Gupta et al. conducted a poll of current practice related to embolisation of PAVMs among 21 HHT experts in nine countries [19]; most of these experts indicated that fewer than one-half of physicians practicing outside of HHT centers recommended embolisation for an asymptomatic PAVM with a feeding artery of at least 3 mm, often resulting in major complications. Our results highlight additional reasons to recommend the procedure for all patients with PAVMs when technically feasible.

Interestingly, the presence of “small” PAVMs was not associated in our study with an impairment in respiratory-related QoL. The impact of small PAVMs in terms of risk of systemic complications is subject to debate. A recent study reported that the presence of small PAVMs, as demonstrated by a grade I shunt on contrast echocardiography, is not associated with an increased risk of systemic emboli [20]. The authors suggested that prophylactic antibiotherapy might not be necessary in these patients. Our results also submit that the presence of small PAVMs might not be deleterious in terms of QoL. Another explanation for our results is that the SGRQ is not sensitive enough to detect small impairments in respiratory-specific QoL.

Our study has several limitations. First, we used the SGRQ to measure respiratory-specific QoL. Our objective was to minimize the impact on the SGRQ score of other clinical manifestations of the disease, such as gastrointestinal bleeding or epistaxis. The SGRQ was developed initially for patients with airway diseases, such as asthma and chronic obstructive pulmonary disease [15]. It has also been used in a variety of respiratory disorders, such as pulmonary fibrosis [21], pulmonary hypertension [22], and chronic pulmonary aspergillosis [23]. It has been validated in the French language [24]. Our results suggest that the SGRQ can also be used in patients with HHT and PAVMs, since it is able to differentiate between different levels of lung disease severity and to detect a beneficial effect of embolisation of PAVMs on health status. Second, the SGRQ score itself can be affected by other manifestations of HHT, such as anemia or cardiac insufficiency secondary to liver disease. However, the level of haemoglobin was similar in the three groups and there was one patient with severe liver disease (i.e., responsible for pulmonary hypertension) in each group. Third, there was no randomization with respect to the decision to perform embolisation, because we felt that it was not ethical not to advise embolisation whenever possible to all patients, as recommended in the international guidelines. However, this was a prospective study and the QoL was evaluated twice in patients who underwent embolisation of PAVMs (immediately prior to the embolisation and approximately 6 mo after); this enabled us to use the subjects as their own controls.

In summary, in patients with HHT, the presence of PAVMs negatively impacts QoL when the size of the feeding artery is large enough to be embolised. Moreover, embolisation of PAVMs normalises respiratory-specific QoL. These results may constitute an additional argument to convince patients to undergo this procedure, not only because it is beneficial in terms of prevention of systemic and cerebral complications, but also because it may increase QoL.
